# *Leptospira* Exposure and Patients with Liver Diseases: A Case-Control Seroprevalence Study

**Published:** 2016-06

**Authors:** Cosme Alvarado-Esquivel, Luis Francisco Sánchez-Anguiano, Jesús Hernández-Tinoco, Agar Ramos-Nevárez, Sandra Margarita Cerrillo-Soto, Carlos Alberto Guido-Arreola

**Affiliations:** 1 Biomedical Research Laboratory. Faculty of Medicine and Nutrition, Juárez University of Durango State, Durango, Mexico;; 2 Institute for Scientific Research “Dr. Roberto Rivera Damm”, Juárez University of Durango State, Durango, Mexico;; 3 Clínica de Medicina Familiar. Instituto de Seguridad y Servicios Sociales de los Trabajadores del Estado. Predio Canoas S/N. 34079 Durango, Mexico

**Keywords:** Leptospira, epidemiology, seroprevalence, liver diseases, case-control study, association, Mexico

## Abstract

The seroepidemiology of *Leptospira *infection in patients suffering from liver disease has been poorly studied. Information about risk factors associated with infection in liver disease patients may help in the optimal planning of preventive measures. We sought to determine the association of *Leptospira *IgG seroprevalence and patients with liver diseases, and to determine the characteristics of the patients with *Leptospira *exposure. We performed a case-control study of 75 patients suffering from liver diseases and 150 age- and gender-matched control subjects. Diagnoses of liver disease included liver cirrhosis, steatosis, chronic hepatitis, acute hepatitis, and amoebic liver abscess. Sera of participants were analyzed for the presence of anti- *Leptospira *IgG antibodies using a commercially available enzyme immunoassay. Anti-*Leptospira* IgG antibodies were found in 17 (22.7%) of 75 patients and in 15 (10.0%) of 150 control subjects (OR = 2.32; 95% CI: 1.09-4.94; *P=*0.03). This is the first age- and gender-matched case control study about *Leptospira* seroprevalence in patients with liver diseases. Results indicate that *Leptospira* infection is associated with chronic and acute liver diseases. Results warrants for additional studies on the role of *Leptospira* exposure in chronic liver disease.

## INTRODUCTION

*Leptospira* is a bacterium responsible of infections in humans and animals all around the world (1, 2). Infection with *Leptospira* causes a disease known as leptospirosis (2). This disease is considered an emerging infectious disease (3), and represents a public health problem particularly in urban centers of developing countries (1). Leptospirosis is a zoonosis (4). *Leptospira* is transmitted to humans by contact with *Leptospira*-infected animals or with environment contaminated with *Leptospira* (5). Skin abrasions represent the main route of *Leptospira* infection (6). Most infections with *Leptospira* are subclinical or may result in a very mild illness without any complication (5). Clinical manifestations of leptospirosis depend upon the predominant organs involved, and the case fatality ratio of leptospirosis could be 40% or higher (5). In mild cases, leptospirosis is presented as a flu-like illness (1). Fever, myalgia, and headache are common clinical manifestations of leptospirosis (6). In severe cases of the disease, patients have liver involvement with jaundice, splenomegaly, and nephritis (Weil’s disease) (5). In addition, infection with *Leptospira* may lead to disease in eyes (7), lungs (8), heart (6), and brain (9).

Liver is commonly involved in leptospirosis (6, 10). Infection with *Leptospira* in humans has been linked to acute hepatitis (11), enlargement of and lesions in liver (12), liver damage (13), and hepatic failure (14). Morphological abnormalities of liver including severe edema with pronounced dissociation of the liver cell bands, cholestasis, cloudy swelling of hepatocytes and necrosis have been reported in leptospirosis (15, 16). The epidemiology of leptospirosis in patients with liver disease is largely unknown. To the best of our knowledge, the magnitude of *Leptospira* exposure in patients with liver diseases in Mexico has not been studied. Therefore, this study aimed to determine the association of *Leptospira *IgG seropositivity and patients suffering from liver diseases attended in a public hospital in Durango City, Mexico. Seroprevalence association with the socio-demographic, clinical, and behavioral characteristics of the patients with liver disease was also investigated.

## MATERIALS AND METHODS

### Selection and description of participants

An age- and gender- matched case-control study was undertaken using residual serum samples from *Toxoplasma gondii* serosurveys in patients with liver disease (17) and general population (18) in Durango City, Mexico. Patients with liver disease (cases) and subjects without liver disease (controls) were compared for the presence of anti-*Leptospira* IgG antibodies. We used a 1:2 ratio of cases and controls. Thus, the group of cases included 75 patients with liver disease, and the group of controls included 150 subjects without liver disease. Patients with liver disease were attended in the Gastroenterology Department of a public hospital (Mexican Social Security Institute) in Durango City, Mexico. Of the 75 patients, 47 were males and 28 females. They were 58.65 ± 14.41 (range: 22-85) years old. Diagnoses of liver disease included liver cirrhosis in 67 patients, steatosis in four, chronic hepatitis in two, acute hepatitis in one, and amoebic liver abscess in one. Liver cirrhosis was caused by alcohol consumption in 35 patients, and by hepatitis C virus in four patients. The etiology of liver cirrhosis was unknown in 28 patients. The control group consisted of 94 males and 56 females matched with cases by age and gender. Control subjects were randomly selected from the general population of Durango City. The mean age in controls was 58.51 ± 14.47 (range: 22-88) years old and comparable with that in patients (*P*=0.94).

### Socio-demographic, clinical, and behavioral data of patients with liver diseases

Data of the patients with liver disease was obtained from previously summited questionnaires (17). Socio-demographic data included age, gender, birthplace, residence, educational level, occupation and socioeconomic status. Clinical data included diagnosis and duration of liver disease, response to treatment, presence of concomitant diseases, history of blood transfusion, organ transplantation, surgery, lymph node enlargement and headache. In addition, information about the presence of impairments in memory, vision, reflexes and hearing was obtained. Liver cirrhosis was diagnosed by clinical manifestations and liver biopsy. Behavioral data included contact with animals, traveling, type of meat consumed, degree of meat cooking, consumption of unwashed raw vegetables or fruits, unpasteurized milk, untreated water, frequency of eating in restaurants or fast food outlets, soil contact, and type of flooring at home.

### Technical information

Serum samples of patients with liver diseases and controls were tested for anti-*Leptospira* IgG antibodies using a commercially available enzyme-linked immunosorbent assay kit, “*Leptospira* IgG ELISA test” (Diagnostic Automation Inc., Calabasas, CA). This test uses purified *Leptospira* Patoc 1 antigen. An absorbance reading of ≥ 0.5 optical density (OD) units was used as a cut-off for seropositivity. Weakly reactive sera were those with absorbance readings between 0.5 and 1.0 OD units. Whereas, strongly reactive sera (or with high antibody levels) were those with absorbance readings > 1.0 OD units. According to the manufacturer’s package insert, this assay has a sensitivity of 100% and a specificity of 100%. All tests were performed following the manufacturer´s instructions. Positive and negative controls included in the kit were used in each run.

### Statistical analysis

Statistical analysis was performed using the software Microsoft Excel 2013, Epi Info version 7 and SPSS version 15.0. Comparison of age between cases and controls was made with the student´s t test. We calculated odds ratios (OR) and 95% confidence intervals (CI) for the association of *Leptospira* seropositivity and liver diseases. The association between patients’ characteristics and *Leptospira* seropositivity was assessed by bivariate analysis. Comparison of the frequencies between groups was made with the Pearson’s chi-squared test and with the two-tailed Fisher exact test (for small values), and a *P* value <0.05 was considered statistically significant.

### Ethical aspects

This project was approved by the Ethics Committee of the Instituto de Seguridad y Servicios Sociales de los Trabajadores del Estado in Durango City. We examined only stored sera and data of participants from previous serosurveys (17, 18). In those surveys, the purpose and procedures of the studies were explained to all participants. Written informed consent was obtained from all participants.

## RESULTS

Anti-*Leptospira* IgG antibodies were found in 17 (22.7%) of 75 patients and in 15 (10.0%) of 150 control subjects. The difference in *Leptospira* exposure between cases and controls was statistically significant (OR = 2.32; 95% CI: 1.09-4.94; *P=*0.03). The frequency of high levels (≥1.0 OD units) of anti-*Leptospira* IgG antibodies in patients with liver disease (2/75: 2.7%) was similar to the one (4/150: 2.7%) in control subjects (*P*=1.00). Stratification by gender showed that the frequency of *Leptospira* exposure in male cases (10/47: 21.3%) was similar to that (10/94: 10.6%) in male controls (*P*=0.12). Whereas, the frequency of *Leptospira* exposure in female cases (7/28: 25.0%) was similar to that (5/56: 8.9%) in female controls (*P*=0.09). None of the 28 female cases and 56 female controls had high levels (≥1.0 OD units) of anti-*Leptospira* IgG antibodies. The frequency of high levels of anti-*Leptospira* IgG antibodies in male cases (2/47: 4.3%) was similar to that (4/94: 4.3%) in male controls (*P*=1.00). All but one seropositive controls were healthy. The ill seropositive control suffered from rheumatism.

None of the socio-demographic characteristics of patients including age, gender, birthplace, residence, educational level, occupation and socioeconomic status was associated with *Leptospira *exposure (*P*>0.05). Concerning clinical characteristics of patients, the frequency of *Leptospira* exposure was similar in patients regardless the diagnosis and duration of liver disease, response to treatment, presence of concomitant diseases, history of blood transfusion, organ transplantation, surgery, lymph node enlargement, headache, and impairments in memory, vision, reflexes and hearing (Table 1). Seropositivity to* Leptospira* was found in 16 (23.9%) of 67 cirrhotic patients, and in one (33.3%) of 3 hepatitis patients. No seropositive cases for *Leptospira* were found in four patients with steatosis or in one patient with amoebic liver abscess. The only one patient with acute hepatitis had low (0.86 OD units) antibody levels against *Leptospira*.

**Table 1:** Bivariate analysis of clinical data and infection with *Leptospira* in patients with liver disease

Characteristic	No. of subjects tested*	Prevalence of *Leptospira* infection		*P* value
		No.	%	

Concomitant disease				
Yes	26	9	34.6	0.08
No	49	8	16.3	
Duration of liver disease				
One or less years	30	5	16.7	0.4
More than one year	45	12	26.7	
Response to treatment				
Good	50	12	24	0.62
Regular	4	1	25	
Bad	3	0	0	
Lymphadenopathy ever				
Yes	11	3	27.3	0.7
No	64	14	21.9	
Headache frequently				
Yes	25	4	16	0.33
No	50	13	26	
Blood transfusion				
Yes	50	12	24	0.69
No	25	5	20	
Transplantation				
Yes	4	1	25	1
No	71	16	22.5	
Surgery ever				
Yes	42	8	19	0.39
No	33	9	27.3	
Memory impairment				
Yes	32	8	25	0.67
No	43	9	20.9	
Reflexes impairment				
Yes	18	5	27.8	0.53
No	57	12	21.1	
Hearing impairment				
Yes	27	5	18.5	0.52
No	48	12	25	
Visual impairment				
Yes	48	12	25	0.52
No	27	5	18.5	

Seroprevalence of *Leptospira* exposure in patients with alcohol-related liver disease (9/35: 25.7%) was similar to that (8/40: 20.0%) in patients with other types of liver diseases (*P*=0.55). With respect to the behavioral characteristics, the frequency of *Leptospira* seropositivity was lower in patients with consumption of pork (13/70: 18.6%) than in those without this practice (4/5: 80.0%) (*P*=0.008). Similarly, the frequency of *Leptospira* seropositivity was higher in patients with consumption of quail meat (2/2: 100%) than in those without this practice (15/71: 21.1%) (borderline association: *P*=0.05). Other behavioral characteristics including contact with animals, traveling, consumption of meat other than pork or quail meat, degree of meat cooking, consumption of unwashed raw vegetables or fruits, unpasteurized milk, untreated water, frequency of eating in restaurants or fast food outlets, soil contact, and type of flooring at home did not show an association with *Leptospira* seropositivity.

## DISCUSSION

Although it is well known that *Leptospira* is a pathogen that causes liver damage, the epidemiology of *Leptospira* infection in patients suffering from liver diseases is largely unknown. We are not aware of any report on the association of *Leptospira *infection with patients with liver cirrhosis or other chronic liver diseases. Therefore, we decided to perform this age- and gender matched case-control study to determine such association in patients with liver disease attended in a public hospital in Durango City, Mexico. We found that seroprevalence of *Leptospira* exposure was significantly higher in patients suffering from liver diseases than in control subjects without liver disease. Results thus indicate that *Leptospira* exposure is associated with liver disease in Durango City. Seroprevalence (22.7%) found in patients with liver diseases is also higher than the 4.4% seroprevalence of *Leptospira* exposure in waste pickers in the same Durango City reported recently (19), and the 15.6% seroprevalence reported in general population of rural Durango (20) using the same enzyme immunoassay. Acute infection with *Leptospira* has been linked with severe liver disease i.e., Weil’s disease (5, 13, 14), and *Leptospira* infection may progress into a chronic, largely asymptomatic infection (21-23). However, very little is known about the clinical picture of chronic infection with *Leptospira* in humans. In the present study, the great majority of patients suffered from a chronic liver disease. Therefore, the association of *Leptospira* exposure found in these patients suggests that *Leptospira* is involved in chronic liver disease. Further studies to determine the clinical features of liver disease in chronic infections with *Leptospira* are needed. Apart from the potential causative role of *Leptospira* infection in liver disease, the association of *Leptospira* exposure in patients with liver diseases may mean that: 1) these patients have behavioral factors that might facilitate infection with *Leptospira*, and 2) other unknown factors are contributing for infection in these patients. It is not clear whether *Leptospira* infection occurred before or during the development of liver disease. Longitudinal studies to evaluate the pathogenic role of *Leptospira* in chronic liver disease patients should be conducted.

We attempted to determine sociodemographic, clinical and behavioral characteristics associated with *Leptospira* exposure in the liver disease patients studied. Bivariate analysis showed that *Leptospira* exposure was negatively associated with consumption of pork. This result means that consumption of pork did not play any contributing role in *Leptospira* infection in patients. On the other hand, a borderline (*P*=0.05) association between *Leptospira* seropositivity and consumption of quail meat was found. A search for this association in the medical literature found no results. We are not aware of any report about the presence of *Leptospira* infection in quails. However, infection with *Leptospira* in quails might be possible since this infection has been reported in birds (24).

Alcohol-related toxicity and alcoholic hepatitis can occasionally produce clinical features similar to leptospirosis (25). It is unclear whether *Leptospira* infection may modify the severity of alcohol-related liver disease or whether these patients may have a more severe *Leptospira* infection. In the present study, patients with alcohol-related liver disease had similar seroprevalence of *Leptospira* exposure to those without this diagnosis.

This study has some limitations. The sample size of liver disease patients was small and only few types of liver diseases among patients were present. In addition, very few subjects were positive to *Leptospira* and therefore statistical power was limited. Further research with larger sample sizes of patients with specific diagnoses of liver diseases to determine their association with *Leptospira* infection is needed.

## CONCLUSIONS

This is the first age- and gender-matched case control study about Leptospira seroprevalence and liver diseases. Results indicate that Leptospira exposure is associated with chronic and acute liver diseases. Results warrants for further studies on the role of Leptospira exposure in chronic liver disease.
